# *PDGFA* gene rs9690350 polymorphism increases biliary atresia risk in Chinese children

**DOI:** 10.1042/BSR20200068

**Published:** 2020-07-21

**Authors:** Fei Liu, Jixiao Zeng, Deli Zhu, Xiaogang Xu, Menglong Lan, Mengmeng Wang, Jinglu Zhao, Huimin Xia, Yan Zhang, Ruizhong Zhang

**Affiliations:** 1 Department of Pediatric Surgery, Guangzhou Institute of Pediatrics, Guangzhou Women and Children’s Medical Center, Guangzhou Medical University, Guangzhou, Guangdong, China; 2 Department of Anesthesiology, Guangzhou Women and Children’s Medical Center, Guangzhou Medical University, Guangzhou, Guangdong, China

**Keywords:** biliary atresia, GWAS, PDGFA, polymorphism, susceptibility

## Abstract

Biliary atresia (BA) is a genetic and severe fibro-inflammatory obliterative cholangiopathy of neonates. Platelet-derived growth factor subunit A (PDGFA), as one of participants in liver fibrosis, the overexpression of *PDGFA* through DNA hypomethylation may lead to the development of BA, but the pathogenesis is still unclear. We conducted a large case–control cohort to investigate the association of genetic variants in *PDGFA* with BA susceptibility in the Southern Chinese population (506 cases and 1473 controls). We observed that the G allele of rs9690350(G>C) in *PDGFA* was significantly associated with an increased risk of BA (OR = 1.24, 95% CI = 1.04–1.49, *P*=0.02). Additionally, the rs9690350 G allele increased the risk of non-cystic biliary atresia (OR = 1.26, 95% CI = 1.04–1.52, *P*=0.02) and was a genetic biomarker of severe manifestations after surgery. These findings indicate that the rs9690350 G allele is a *PDGFA* polymorphism associated with the risk of BA that may confer increased disease susceptibility.

## Introduction

Biliary atresia (BA) is a common and serious pediatric surgical disease characterized by progressive fibrosis of the intrahepatic and extrahepatic bile ducts. The detailed mechanisms of BA is remain uncertain, but may be correlate with genetic defects [1], viral infections [2,3], and/or immune disorders [4]. Kasai portoenterostomy is the first line treatment for BA, which aims to restore bile drainage and alleviate cholestasis. However, most children eventually need liver transplantation because of progressive liver fibrosis [5]. It has been reported that the long-term survival rates of patients with BA in America are 13–50% [6]. Considering the poor prognosis of BA, further exploration of its etiology are required to identify effective therapeutic targets.

Hepatic progressive fibrosis is an important pathological feature of BA, characterized by the accumulation of extracellular matrix (ECM) following chronic inflammatory liver injury. A central event in the pathogenesis of hepatic fibrosis is the activation and proliferation of hepatic stellate cells (HSCs) and their transformation into myofibroblasts [5]. The activation of HSCs can be triggered by various growth factors, including mitogen platelet-derived growth factor (*PDGF*), epidermal growth factor (*EGF*), and fibrogenic transforming growth factor-β (TGF-β) [6]. *PDGF* is currently regarded as the most important mitogen for HSC activation during hepatic fibrogenesis [5,7] and BA [8].

To date, four PDGF subunits (PDGF-A, -B, -C, and -D) have been identified that dimerize through disulphide linkages to form homodimers (AA, BB, CC, and DD) or heterodimers (AB) [9,10]. PDGFs regulate an array of cellular functions, including cell proliferation, differentiation, cytoskeletal rearrangements, and cell migration [11,12]. Moreover, PDGFs induce proliferative and fibrotic pathologies in multiple organs, including the skin [13], lung [14], liver [15], kidney [16], and heart [17]. Ahmed et al. showed PDGFA strongly expressed in bile duce cells, suggested that PDGF-A played an important role in the fibrogenesis of biliary atresia [18]. Cofer et al. proposed that DNA hypomethylation mediated the overexpression of genes associated with BA and identified PDGF-A as a novel candidate gene [8].

The PDGF-A promoter contains numerous CpG motifs suggesting its transcription may be regulated through DNA methylation [10]. Given these findings, we hypothesized that SNPs in this region regulate PDGF expression, confirming its role in BA pathogenesis. To test this hypothesis, we performed a case-controlled study with 506 cases and 1473 controls to investigate the relationship between *PDGFA* SNPs and the occurrence of BA.

## Materials and methods

### Study subjects

The present study included a total of 1979 Chinese subjects (Supplementary Table S1), all of whom were enrolled at the Guangzhou Women and Children’s Medical Centre from January 2005 to April 2016. Among these subjects, 506 patients were diagnosed with BA, including 292 males and 214 females, with their mean age as 2.088 ± 1.934 months (range: 1–7 months). The diagnostic criteria of BA includes: (1) jaundice lasting longer than the first 2 weeks of life, acholic stools, dark urine and hepatomegaly; (2) direct bilirubin fraction is greater than 20% of the total as well as an elevated of alanine aminotransferase, aspartate aminotransferase and γ-glutamyl transpeptidase; (3) shrunken gallbladder and no common bile duct is visible in ultrasound examination. The diagnosis of BA was finally confirmed by operative cholangiography. Associated congenital malformations were found in none of these patients. Patients with acute cholangitis and cardiovascular or other diseases that cannot tolerate surgery were excluded from the present study.

Meanwhile, the present study randomly selected a group of 1473 healthy, unrelated gender- and age-matched subjects as a control group (967 males, 506 females). All of these individuals had no history of liver or autoimmune disease and never undergone liver transplantation. The present study got the approval from the Institutional Review Board of Guangzhou Women and Children’s Medical Centre. Medical histories of the participants in the present study were collected during their first routine visit. Informed written consent was acquired from their parents or guardians and the research was conducted in compliance with the World Medical Association Declaration of Helsinki.

### Polymorphism analysis

Using the TIANamp Blood DNA Kit (TianGen Biotech Co. Ltd., Beijing, China) and following the manufacturer’s instructions, the Genomic DNA was generally extracted from 2 ml of a peripheral blood sample. Its concentration and purity were determined using a UV spectrophotometer (Nano Drop Technologies, Inc., Wilmington, DE) by the spectrophotometric method of absorbance at 260 and 280 nm. Briefly, qualified DNA samples were diluted to 10 ng/μl and loaded in 96-well plates.

We searched for potentially regulatory SNPs using the GTEX tool in *PDGFA.* SNP rs9690350 (chr7:508163G>C, GRCh38 genome build) was selected for the further query based on the high confidence of which may play as an expression quantitative locus (eQTL) with *PDGFA* across different tissues in human, especially in Central nervous system (Supplementary Figure S1). We also scored the role of rs9690350 polymorphism using Regulome DB, showing the high probability of this SNP as a regulatory player for this gene (Supplementary Table S2). Using the MassARRAY iPLEX Gold system (Sequenom), a customized multiplex genotyping panel was successfully designed for the SNP (rs9690350 C/G). Four positive controls and four negative controls were included in each of the 384-well plates to ensure the accuracy of the genotyping results. Deviation of the controls from the Hardy–Weinberg equilibrium was tested and all passed with *P*-values > 0.05 (*P*=0.58). The assays were repeated for 5% of the samples, and the results were 100% concordant.

### Statistical analysis

We utilized the *χ*^2^ test to evaluate the differences in the frequency distributions of the demographics and genotypes between BA cases and controls, applied PLINK software to assess the relationship between rs9690350 and BA risk, used odds ratios (ORs) and 95% confidence intervals (CIs) to assess the correlations between the *PDGFA* gene SNP (rs9690350 C/G) and BA susceptibility. Considering *P* values<0.05 was statistically significant.

The association of rs9690350 with disease risk was corrected by logistic regression using age and sex as covariates, and the obtained associations remained significant after all corrections. Cases with a certain subtype, cases without the subtype and healthy controls were compared to analyze the associations between the rs9690350 polymorphism and the BA subtype. Among the cases with and without the subtype, a heterogeneity test for three ORs was compared.

## Results

### Population characteristics

The demographic features of the participants are shown in Table 1, 506 cases and 1473 controls were successfully genotyped in the present study. The genotype distributions of rs9690350 in the case–control data set were in accordance with the Hardy–Weinberg equilibrium (*P=*0.58). Regarding the subtype of BA, 44 patients (8.7%) had CBA and 462 (91.3%) did not.

**Table 1:** Risk allele (G) frequency of *PDGFA* gene SNP (rs9690350) in the case-control data set of children with BA

CHR	SNP	BP	Risk allele	Genetic model	Case No. (F_A)	Control No. (F_U)	OR	95% CI	P*
7	rs9690350 (HWE = 0.58)	508163	G	**Allelic**	**600/314 (0.66)**	**1824/1059 (0.63)**	**1.24**	**(1.04–1.49)**	**0.02**
				Dominant	399/58 (0.87)	1246/196 (0.86)	1.21	(0.84–1.73)	0.31
				Recessive	201/256 (0.44)	579/863 (0.40)	1.38	(1.08–1.76)	0.01
				Genotypic	–	–	–	–	0.04

Abbreviations: BA, biliary atresia; BP, base pair (where the SNP is located); CI, confidence interval; CHR, chromosome; F_A/F_U, risk allele frequency of the SNP in cases or controls; HWE, Hardy–Weinberg equilibrium; OR, odds ratio; SNP, single-nucleotide polymorphism.

P* adjusted by gender and age. The *P-*value indicates the significance based on allelic association tests. Calculation of the OR was also based on the risk allele of each SNP.

### Associations between *PDGFA* rs9690350 polymorphism and BA risk

Compared with normal control group subjects (Table 1), we found that the G allele frequency of rs9690350 was significantly higher in children with BA (OR = 1.24, 95% CI = 1.04–1.49, *P* =0.02). Similar results were also found using recessive (REC; OR = 1.38, 95% CI = 1.08–1.76, *P*=0.01) and genotypic (GENO) models (*P*=0.04). These results suggest that the rs9690350 G allele is a risk allele for increased susceptibility to BA.

### Associations between rs9690350 polymorphism and the BA subtype

We explored the relationship between rs9690350 and the BA subtype based on the context reported by previous studies that patients with cystic biliary atresia (CBA) have a better clinical outcome after a hepaticojejunostomy (Table 2) [19,20]. All of the CBA cases were confirmed by cholangiography. We found that the rs9690350 G allele was significantly associated with an increased risk of non-CBA (OR = 1.26, 95% CI = 1.04–1.52, *P*=0.02). Similar results were found in the REC model (OR = 1.34, 95% CI = 1.04–1.73, *P*=0.02). However, we failed to observe any association with rs9690350G allele and CBA susceptibility (OR = 0.94, 95% CI = 0.58–1.52, *P*=0.79), which may affect by either the limited sample size for the CBA patients or the association of *PDGFA* with disease that was more likely to affect the severe cases (Table 3). Further experiments are required to further investment.

**Table 2:** The results of *PDGFA* gene SNP (rs9690350) and non-CBA patients

CHR	SNP	BP	Risk allele	Genetic model	Case No. (F_A)	Control No. (F_U)	OR	95% CI	*P*
7	rs9690350	508163	G	**Allelic**	**545/277 (0.66)**	**1825/1059 (0.63)**	**1.26**	**(1.04–1.52)**	**0.02**
			G	Dominant	365/46 (0.89)	1246/1196 (0.86)	1.34	(0.91–1.97)	0.13
			G	Recessive	180/231 (0.44)	579/863 (0.40)	1.34	(1.04–1.73)	0.02

Abbreviations: BP, base pair (where the SNP is located); CBA, cystic biliary atresia; CHR, chromosome; CI, confidence interval; F_A/F_U, risk allele frequency of the SNP in cases or controls; OR, odds ratio; SNP, single-nucleotide polymorphism.

The *P* value indicates the significance based on allelic association tests. Calculation of the OR was also based on the risk allele of each SNP.

**Table 3:** The results of *PDGFA* gene SNP (rs9690350) and CBA patients

CHR	SNP	BP	Risk allele	Genetic model	Case No. (F_A)	Control No. (F_U)	OR	95% CI	*P*
7	rs9690350	508163	**G**	**Allelic**	**48/30 (0.62)**	**1825/1059 (0.63)**	**0.94**	**(0.58–1.52)**	**0.79**
			G	Dominant	29/10 (0.89)	1246/196 (0.86)	0.48	(0.22–1.03)	0.13
			G	Recessive	19/20 (0.44)	579/863 (0.40)	1.84	(0.95–3.59)	0.07

Abbreviations: BP, base pair (where the SNP is located); CBA, cystic biliary atresia; CHR, chromosome; CI, confidence interval; F_A/F_U, risk allele frequency of the SNP in cases or controls; OR, odds ratio; SNP, single-nucleotide polymorphism.

The *P* value indicates the significance based on allelic association tests. Calculation of the OR was also based on the risk allele of each SNP.

## Discussion

Progressive hepatic fibrosis is one of the major pathological features of BA and is associated with ECM accumulation and HSC activation. The etiology of BA remains unclear, possibly due to gene polymorphisms. PDGF, as a well-recognized mitogen for HSCs and an important fibrogenic factor in chronic human liver disease, plays a pivotal role in the development of hepatic fibrosis. Several studies showed that implement strategies aim at blocking PDGF signaling can ameliorate liver fibrogenesis [21,22]. In the present study, we have shown for the first time that the PDGFA gene SNP (rs9690350), particularly the G allele, mediate BA pathogenesis through increased PDGFA expression.

PDGF plays an important role in liver fibrogenesis through the transdifferentiation and proliferation of HSCs/myofibroblasts, in addition to induction of collagen deposition. Although all PDGF isoforms can induce liver fibrosis [7,11], previous studies have principally focused on PDGF-BB and its receptor PDGFR-β. These proteins have received intense research interest due to their potentially prominent roles in liver fibrogenesis. Czochra et al*.* found that the expression of PDGF-B and its receptors increased in both experimental fibrosis rats and human fibrotic livers [23]. Grappone and co-workers reported that PDGF-BB and B chain mRNA expression increased for up to 28 days in the epithelial cells of proliferating bile ducts and periductal mesenchymal cells, suggesting that PDGF-BB contributes to fibrogenesis *in vivo* [24]*.*

PDGFA, as a member of the PDGF family, is involved in organic fibrosis, not only in the liver but also in other organs, including the heart [9], lungs [25], and kidneys [12]. Florian et al*.* [26] showed that PDGFA overexpression in the liver of transgenic mice was accompanied by significant increases in hepatic procollagen III mRNA and TGF-β1 expression, indicating that PDGFA contributes to hepatic fibrosis through TGF-β1 signaling. In addition, PDGFA was expressed in activated HSCs, with the smallest changes in expression observed during hepatic fibrogenesis [27]. Few studies on the relationship between PDGFA and BA hepatic fibrosis have been reported. Recently, Cofer et al*.* [8] revealed that *PDGFA* hypomethylation is involved in the pathogenesis of BA by mediating its overexpression in liver explants. However, this analysis failed to identify the specific methylation changes in the *PDGFB* gene. We considered that the differences might be due to the expression of alternative PDGF isoforms during hepatic fibrosis, i.e., that PDGF-B may be the dominant isoform at the initial stages of hepatic fibrosis, while PDGFA may predominate during later fibrosis stages due to the effects of hypomethylation [27]. Our immunohistochemistry results were consistent with this scenario, with the PDGFA expression observed to progressively increase during disease development.

The human *PDGFA* gene is located on chromosome 7p22, and the promoter region contains numerous CpG motifs, suggesting its potential to be regulated at the transcriptional level by DNA methylation [10,28]. Kaetzel et al*.* [10] identified a significant increase in *PDGFA* mRNA levels in HeLa cells using methyltransferase inhibitors, suggesting that DNA methylation plays a pivotal role in developmental patterns of *PDGFA* gene transcription. Moreover, many SNPs are associated with changes in CpG island methylation, and may promote disease occurrence and progression [29,30]. The present study identified significant differences in the frequency of *PDGFA* gene SNP rs9690350 between BA and control groups, suggesting that rs9690350, particularly the G allele, may be involved in BA pathogenesis. In addition, the rs9690350 G allele was associated with increased BA risk in non-CBA patients, indicating that that allele may affect the occurrence of BA subtypes. The specific mechanism of this effect requires further investigation.

This was the first validation of the association between *PDGFA* gene polymorphisms and BA susceptibility in South Chinese children. Some potential limitations should be addressed. First, only 506 patients (including 44 CBA cases) were included. Although this was the largest study cohort for this condition in Chinese children, the relatively small sample size may have reduced the statistical power of the study. Second, we only investigated a single polymorphism (rs9690350) in the *PDGFA* gene. Additional polymorphisms, particularly functional SNPs, should be considered in further studies. Finally, the present study was restricted to Chinese Han subjects from Southern China, and the results should only be extrapolated to other ethnic groups with caution.

In summary, the present study was the first to describe the relationship between the *PDGFA* SNP rs9690350 and BA pathogenesis. Other PDGF isoforms and their functions in BA should now be studied. Based on these data, PDGFA can be regarded as a potential biomarker for the diagnosis of BA.

## Supplementary Material

Supplementary Figures S1-S2 and Table S1

## Data Availability

The data used to support the findings of this study are included within the article.

## Abbreviations

BA: biliary atresia
CBA: cystic biliary atresia
CI: confidence intervals
ECM: extracellular matrix
EGF: epidermal growth factor
eQTL: expression quantitative locus
HSC: hepatic stellate cell
OR: odds ratio
*PDGF*: platelet-derived growth factor
TGF-β: transforming growth factor-β
