# Supplementary material for: PDGFA gene rs9690350 polymorphism increases biliary atresia risk in Chinese children
Source: Biosci Rep. 2020 Jul 21;40(7):BSR20200068. doi: 10.1042/BSR20200068 (PMC7374268; doi:10.1042/BSR20200068)
Supplement: Supplementary Figures S1-S2 and Table S1 [file BSR-2020-0068_supp.pdf]

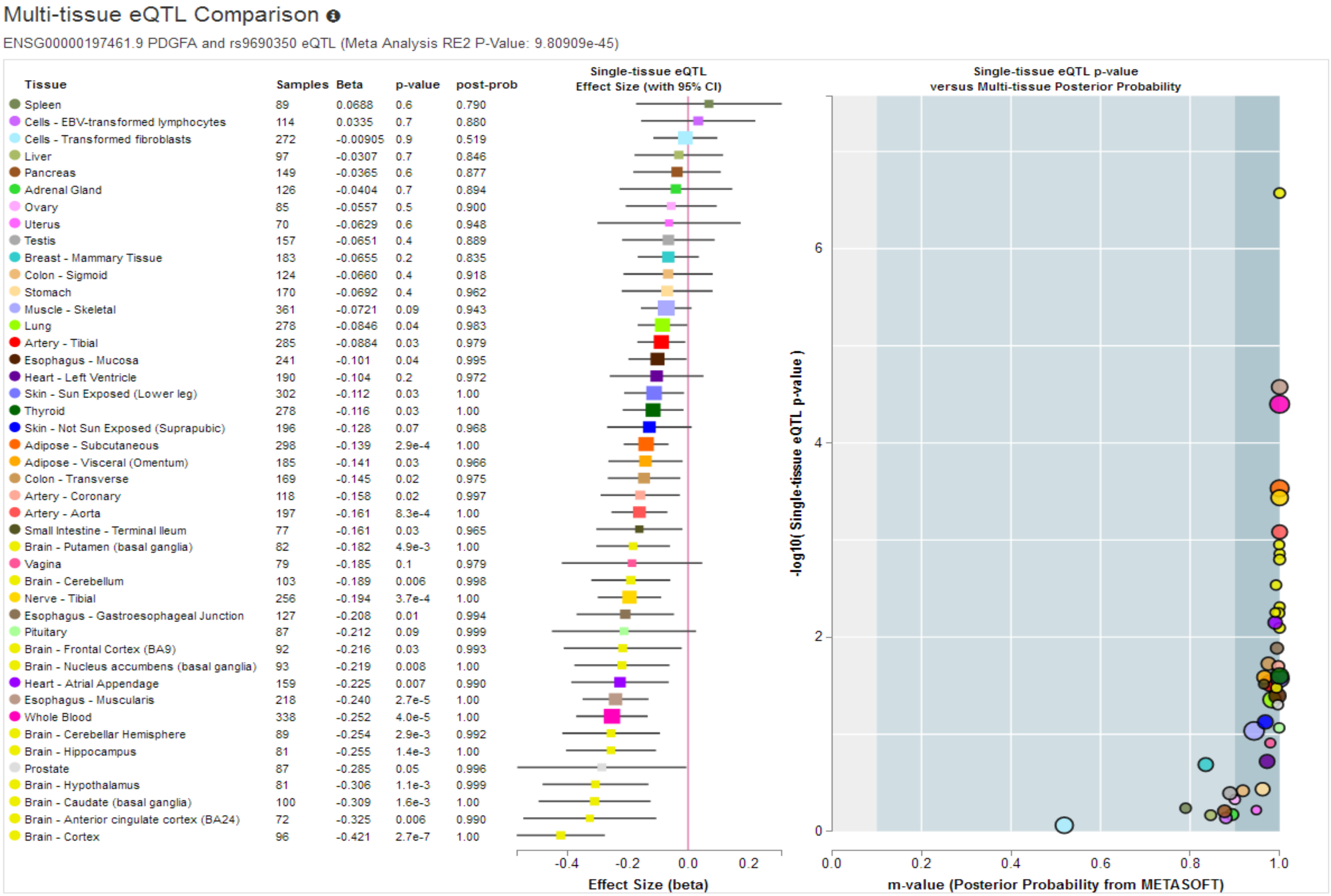

Supplementary Figure 1. SNP rs9690350 played as an expression quantitative locus (eQTL) with *PDGFA* across different tissues in human, especially in Central nervous system using GTEX tool (https://www.gtexportal.org/home/).

Supplementary Table 1. The detailed clinical information of the subjects in this study

Variable	Cases (n=506)	%	Controls (n=1473)	%
Age (Months)	2.088±1.934		18.61±19.75	
≤3	470	92.89%	521	35.37%
>3	36	7.11%	952	64.63%
Gender				
Females	214	42.29%	506	34.35%
Males	292	57.71%	967	65.65%
Clinical manifestation				
Cystic biliary atresia (CBA)	44	8.70%		
non-CBA	462	91.30%		

Supplementary Table 2. The functional annotation of candidate SNP rs9690350 by SCAN (http://www.scandb.org/newinterface/index_v1.html) and Regulome DB (http://regulomedb.org/)

SNP	CHR	A1/A2	SCAN				Regulome DB	
			gene	feature	left_gene	right_gene	hits	score
rs9690350	7	C/G	PDGFA	intron[NM_033023.3]	*LOC100132080*	*PRKAR1B*	TF binding + any motif + DNase Footprint + DNase peak	**2b***
